# The rate sensitivity and plastic deformation of nanocrystalline tantalum films at nanoscale

**DOI:** 10.1186/1556-276X-6-186

**Published:** 2011-03-01

**Authors:** Zhenhua Cao, Qianwei She, Yongli Huang, Xiangkang Meng

**Affiliations:** 1 National Laboratory of Solid State Microstructures, Nanjing University, Nanjing 210093, People's Republic of China; 2 Department of Material Science and Engineering, Nanjing University, Nanjing 210093, People's Republic of China; 3 Key Laboratory of Low Dimensional Materials and Application Technology of Ministry of Education, Faculty of Materials and Photoelectronics Physics, Xiangtan University, Xiangtan 411105, People's Republic of China

## Abstract

Nanoindentation creep and loading rate change tests were employed to examine the rate sensitivity (*m*) and hardness of nanocrystalline tetragonal Ta films. Experimental results suggested that the *m *increased with the decrease of feature scale, such as grain size and indent depth. The magnitude of *m *is much less than the corresponding grain boundary (GB) sliding deformation with *m *of 0.5. Hardness softening behavior was observed for smaller grain size, which supports the GB sliding mechanism. The rate-controlling deformation was interpreted by the GB-mediated processes involving atomic diffusion and the generation of dislocation at GB.

## Introduction

Much research interest has been focused on uncovering the novel plastic deformation mechanisms of nanocrystalline (NC) metals over the last two decades [1-5]. As the average grain size (*d*) decreases to less than 100 nm, grain boundary (GB)-mediated processes, such as GB diffusion and sliding, become increasingly more important during plastic deformation [6]. Molecular dynamic simulation [1], bubble raft model [2], and experimental results [3] suggested that the corresponding critical *d *of NC Cu and Ni for softening behavior is below 20 nm. In contrast, the other experimental observations suggest that the strength induced by dislocation activation still increases even if *d *decreases to 20 nm [7,8]. So far, the dominant deformation mechanism of NC metals has not been clear yet.

Strain rate sensitivity (*m*) is an important dynamic parameter for understanding the plastic deformation of polycrystalline metals. In general, NC metals show a higher *m *than that of coarse grain (CG) and ultrafine grain (UFG) counterparts due to the enhanced GB-mediated process. For NC Cu of *d *~ 10 nm, the value of *m *~ 0.06 was ten times higher than that of CG Cu and single grain Cu [9]. A higher *m *of 0.14 was reported for NC Cu with *d *~ 26 nm produced by electric brush plating [10]. NC Ni also exhibited a higher *m *than that of CG and UFG Ni during depth-sensing indentation and tensile testing [11]. The increased *m *was attributed to GB mediated process instead of dislocation activation. In addition to *d*, it was found that the decreasing twin thickness could also increase the *m *of NC metals [12]. In exceptional case, a negative *m *was observed for some nanostructured Al alloy which was caused by the interaction between dislocations and solutes [13]. Recently, it was found that monometallic NC tetragonal Ta also exhibited negative *m *during indentation deformation [14]. The main reason was believed to be the phase transformation underneath the indenter. However, the negative *m *of NC tetragonal Ta was not demonstrated further by subsequent research. In our previous study [15], a remarkable diffusion creep behavior has been revealed for NC tetragonal Ta at room temperature (RT). Nevertheless, the rate-controlling mechanism is still not clear. The aim of this study is to reveal the rate-controlling deformation mechanism of NC tetragonal Ta films by nanoindentation.

## Experimental method

Ta films of two different *d *were deposited on Si (111) substrates in an inert environment of Ar gas by DC magnetron sputtering using a 99.95% pure Ta target. Before deposition, the Ta target was cleaned by sputtering Ar for 30 min. All the substrates were sequentially cleaned in an ultrasonic bath of acetone and alcohol. The base and working pressure of the chamber were kept at 6.0 × 10^-5 ^and 1.4 Pa, respectively. The sputtering power was maintained at about 250 W. During deposition, the growth rate was 45 nm/min. By adjusting the total time of deposition, the thickness of the films was kept at about 2 μm. Different temperatures of the substrate at 300 K RT and 673 K were used for adjusting the grain size of Ta. The microstructure of Ta films was characterized by X-ray diffraction (XRD) using Cu Kα radiation source and transmission electron microscopy (TEM; JEM-2100).

Nanoindentation tests were performed at RT using a TriboIndenter from Hysitron Inc., Minneapolis, MN, USA, with a Berkovich diamond indenter with nominal tip having a radius of curvature *R *of 150 nm. Hence, the minimum depth for self-similar indentation was estimated to be 9 nm, which was calculated from the equation *R*(1 - sin 70.3°) = 0.06*R *[16]. Displacement and load resolution of the instrument were 0.1 nm and 100 nN, respectively. The indentation depth (*h*) was controlled below 1/10 of the film thickness to eliminate the substrate effect. In order to ensure the credibility of the measurements, the drift measurement was performed immediately before testing. Then, the drift rate was calculated by linear regression of the displacement versus time during the drift analysis. The rate was used for correcting the indentation test data. For creep testing, the specimens were first loaded to a peak load (500-9000 μN) at a constant loading rate  μN/s, and then the peak load was held constant for 40 s. Subsequently, the samples were unloaded to 10% of the maximum load and held at the same constant load for thermal drift correction. Apart from creep testing, the samples were measured with maximum load of 9800 μN at different constant loading rates ranging from 1 × 10^-2 ^to 1 × 10^0^/s without holding. Finally, the indenter was withdrawn to zero load. For consistent results, indentation tests at each load were repeated for at least ten times.

## Results and discussion

**Figure 1:**
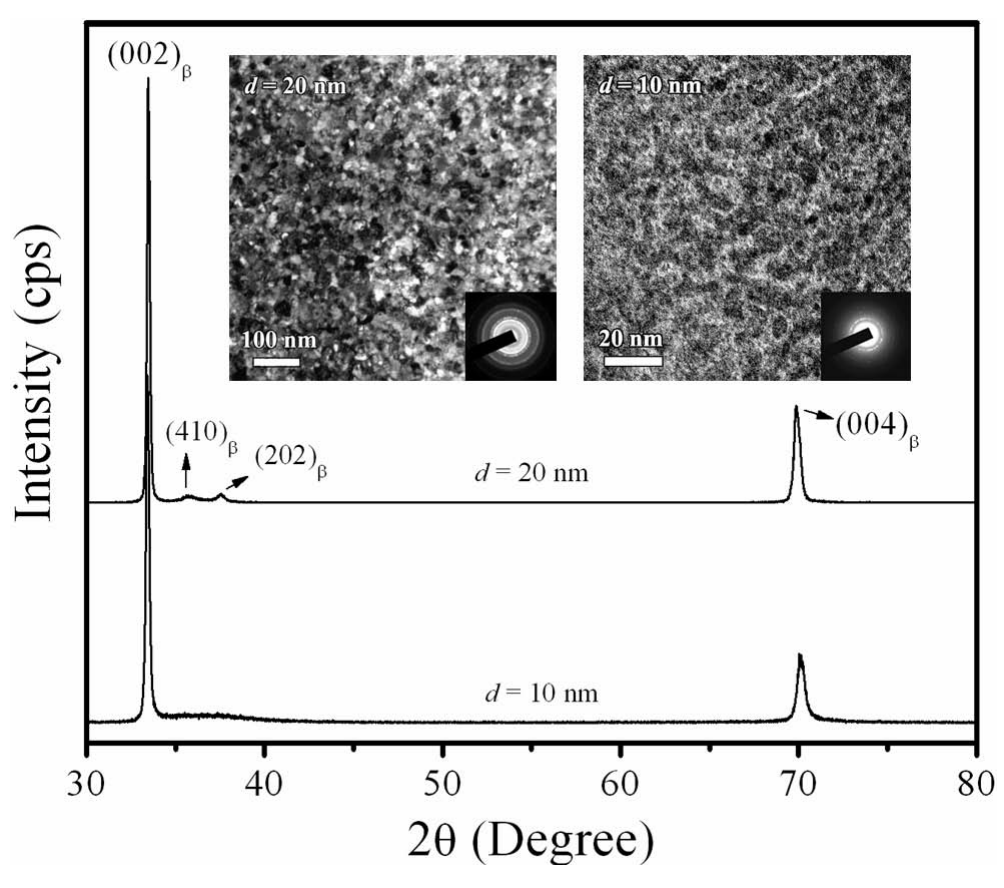
**XRD patterns of the Ta films with different values of *d***. The insets are the bright-field TEM images and the corresponding selected area electron diffractions of the Ta films.

The XRD patterns of tetragonal Ta films are shown in Figure 1. The (002) and (004) diffraction peaks of β phases at 33.6° and 70.8° are found in Ta film prepared at RT. As the sputtering temperature increases to 673 K, the (002) and (004) peaks becomes more intensive, and two more peaks are observed in β phase at (410) and (202), while no peak is observed in α phase. This indicates that the samples consist of almost 100% β phase. It is noted that the two Ta films are not crystalline enough. The value of *d *determined by XRD and TEM is in the range of nanoscale. Even though the sputtering temperature reaches 673 K, the *d *is 20 nm, since the melting point of Ta is as high as 3269 K [17]. In Figure 1, full widths at half maximum (FWHM) of (004) peaks of Ta films are found to be very large. The FWHM of (002) peaks is smaller than that of (004) peaks, because (002) is the main crystal plane for XRD. The results of this study are consistent with those previously reported by Zhang et al. [18]. The plan-view microstructures of tetragonal Ta film with sputtering temperature of 300 and 673 K are shown in TEM counterparts of Figure 1. The corresponding selected area electron diffraction is shown at the right bottom corner of TEM insets. It is found that the grain size distribution is very uniform. The average *d *of the two samples is estimated to be about 10 and 20 nm through TEM images, respectively. It is well known that Scherrer equation is expressed by *d *= *kλ*/(*β*cos *θ*), where *k *is a constant (*k *= 0.9), *λ *is the wave length of the incident X-ray (*λ *= 0.15418 nm for Cu Kα radiation source), *θ *is Bragg angle, and *β *is the FWHM of the diffraction peak [19]. The values of *β *of the Ta films with sputtering temperature of RT and 673 K are 0.031 and 0.019, respectively. The grain sizes determined by Scherrer equation are about 13 and 23 nm, which are in agreement with TEM results.

**Figure 2:**
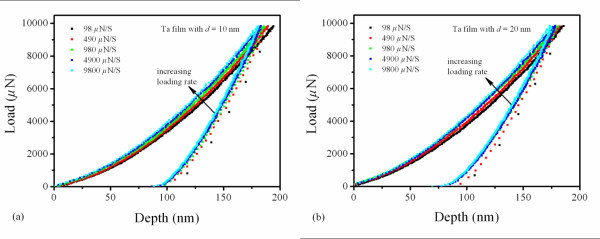
**Load-depth curves at different loading rates for the Ta films with different *d*; a *d *= 10 nm and b *d *= 20 nm**.

**Figure 3:**
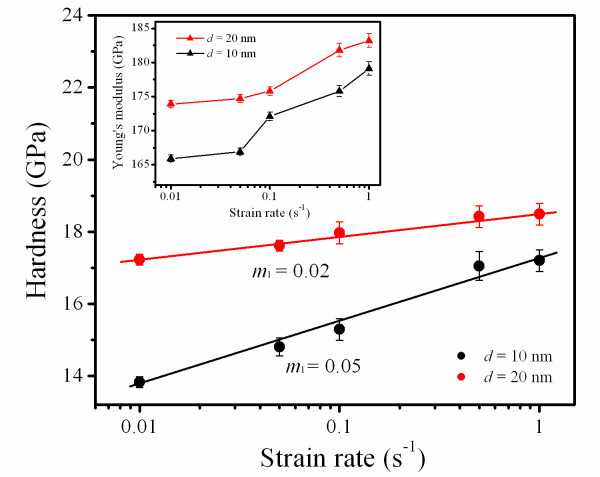
**Hardness versus strain rate of Ta films with *d *of 10 and 20 nm**. The *m*_l _is determined from the slope of the lines. The inset shows the Young's modulus versus strain rate of Ta films with different values of *d*.

It is useful to obtain the effect of strain/loading rate on the mechanical response in revealing the deformation mechanism of NC metals. The variations of load-depth curves of NC Ta films of *d *= 10 and 20 nm with loading rate change are shown, respectively, in Figure 2a,b. Five different loading rates were performed for the rate change testing. With the increased loading rate, in both cases as shown in Figure 2a, b, a higher indentation force is required to impose the same displacement. The influence of loading rate on mechanical response becomes more remarkable for Ta films with a smaller *d *of 10 nm. This suggests that the reduced *d *can enhance the rate sensitivity of NC Ta films. The applied indentation forces become much lower for a smaller *d *at a given depth, which means Ta film with *d *of 10 nm is of lower hardness. The hardness is determined by means of the Oliver-Pharr method [20]. The inset in Figure 3 shows the change of Young's moduli (*E*) with the strain rate. It is found that *E *is directly proportional to *d*. As a result, *E *increases with *d*. These values are slightly smaller than that of NC tetragonal Ta film with larger *d *of 32.3 nm reported by Zhang et al. [18]. It is believed that the stiffness of GB is lower than that of grain interior. The decreased *E *may be associated with the increased GB volume corresponding to decreasing *d *[21,22]. In addition, as strain rate increases, *E *increase in Ta films. The rate-sensitive modulus is contrary to that of NC Au films reported by Jonnalagadda et al. [23]. The elastic deformation usually encompasses both elastic and anelastic behaviors, where the anelastic behavior arising from atomic reconfigurations is time dependent on a much longer scale, i.e., rate-dependent behavior [24]. The GB-mediated process involving atomic diffusion and dislocation generation results in the anelastic behavior, which should be responsible for the rate-sensitive modulus.

Hardness versus strain rate is plotted in Figure 3. In both cases of *d *= 10 and 20 nm, the hardness increases with the enhanced loading rate. Moreover, all the plotted points of *d *= 10 nm show hardness lower than that of *d *= 20 nm in Ta film. It is suggested that a softening behavior occurs as *d *decreases to 10 nm. The loading rate sensitivity (*m*_l_) related to the thermally activation deformation behavior was examined by the definition of , where *H *and  are the hardness and strain rate, respectively [25]. The resultant *m*_l _of Ta films with *d *of 10 and 20 nm are 0.05 and 0.02, respectively. As a result, it is concluded that the magnitude of *m*_l _increases with the decrease of *d*.

**Figure 4:**
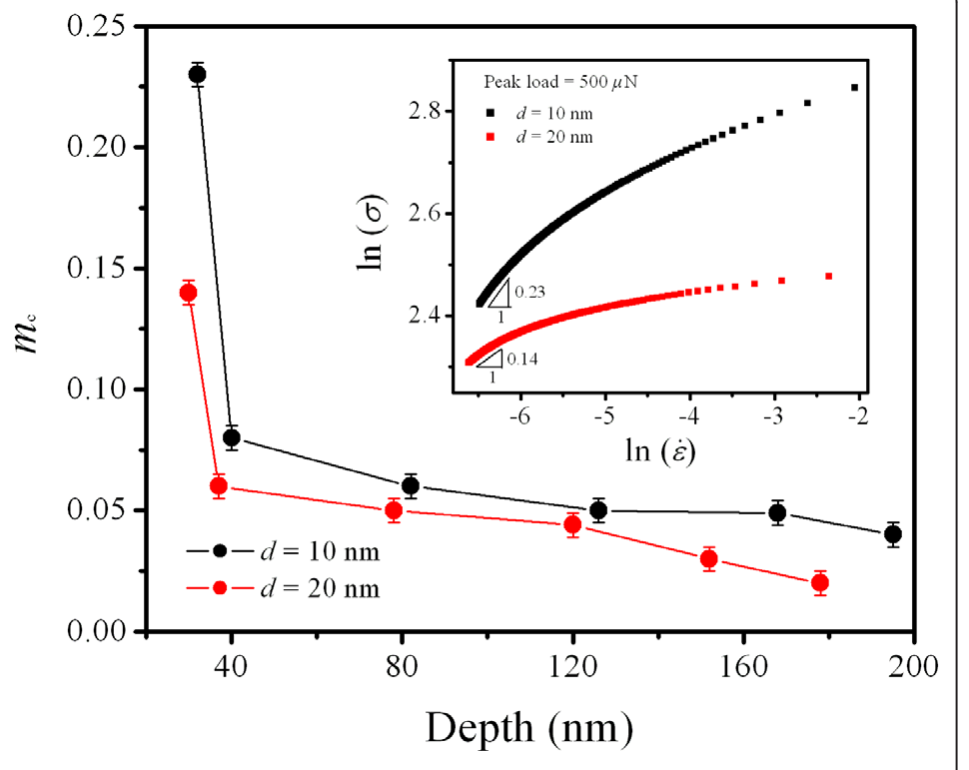
**The *m***_**c **_**versus indent depth for Ta films with *d *values of 10 and 20 nm**. The inset presents the relation between ln(*σ*) and ln() at the peak load of 500 μN.

In addition to *m*_l_, the creep strain rate sensitivity (*m*_c_) was also determined from indentation creep testing. The relation of ln (*σ*) versus ln() at peak load of 500 μN is plotted in the inset of Figure 4, where *σ *is indentation stress. The *m*_c _can be determined by obtaining the slope of the curves. The corresponding procedure is mentioned in our previous study [15]. The *m*_c _of Ta films at different values of *h *is shown in Figure 4. The *m*_c _increases with the decreasing *h *at nanoscale, especially at *h *less than about 80 nm, which exhibits an indentation size effect. The diffusion along tip/sample interface process is believed to be responsible for *h*-dependent *m*_c_. The diffusion path along the tip/sample interface depends on *h*, and it becomes weaker with the increasing *h*. This is consistent with the variation of the indent depth-dependent rate sensitivity. Moreover, the magnitude of *m*_c _when *d *= 10 nm of Ta film is much higher than that when *d *= 20 nm.

It should be noted that the values of both *m*_l _and *m*_c _are positive, which is different from the negative *m *of NC tetragonal Ta as reported by Wang et al. [14]. The negative *m *is attributed to β-α phase transformation underneath the indents. This negative *m *mainly occurs as the loading rate is below the 200 μN/s. However, in this research, most of the loading rates are higher than the 200 μN/s which may induce positive *m*. Grain refinement can often enhance *m*, especially when *d *decreases to nanoscale [9]. The density of GB will significantly increase as *d *decreases to less than below 30 nm. The volume percentage of GB is estimated as GB vol% = 100% - (*d *- *d*_GB_)^3^/*d*^3^, where the *d*_GB _is the thickness of GB [11]. So far, it is a controversy question with respect to accurate determination the thickness of GB in NC metals. Ranganathan et al. [26] estimated that the GB region is only about 0.5 nm wide of the order of two to three lattice plane spacing, while the GB thickness of about 1 nm was reported by Meyers et al. [27]. In ref. [11], it is suggested that the thickness of GB is about seven lattice parameters. Thus, the thickness of GB of tetragonal NC Ta was calculated to be about 3.7 nm. In this study, considering the three values calculated above, we selected an average value of about 2 nm as the thickness of GB for tetragonal NC Ta. Considering the value *d*_GB _= 2 nm, the volume percentages of GB at *d *of 10 and 20 nm are estimated to be about 48.8 and 27.1 vol%, respectively. The enhanced GB density usually advances GB-mediated process, such as Coble creep and GB sliding. However, both *m*_l _and *m*_c _are much lower than *m *= 0.5 expected for diffusion-controlled Coble creep, and *m *= 1 for GB sliding mechanism [28,29]. Hence, GB diffusion and sliding are ruled out as dominant deformation for the present NC Ta films.

The dislocation-mediated mechanism is thus considered as the rate-controlling deformation process. It is well known that dislocation pile-up at GB is responsible for the grain refinement-induced hardening on CG and UFG metals, as they exhibit a normal Hall-Petch relation [30]. However, the resultant hardness decreases as *d *decreases from 20 to 10 nm. Therefore, the dislocation pile-up process is also excluded as the dominant deformation mechanism. The reduction in hardness is due to *d *in support of GB-mediated process, while the low *m*_l _and *m*_c _relative to the Coble creep and GB sliding process with a higher *m *challenges the GB diffusion and sliding mechanism. It seems that there is an inconsistent conclusion obtained from the resultant hardness and the rate sensitivity. It has been documented that the transitional Frank-Read source inside the grain for dislocation nucleation and multiplication becomes invalid since the stress for their operation is inversely proportional to the size of the sources, as the *d *decreases to nano- and submicron-scale [31]. Instead, the GB can be treated as the source of the dislocation emission and nucleation which was demonstrated by TEM observation and MD simulation [32,33]. The dislocation emission is a rate-controlled process which could be thermally activated from GB as the dislocation activation is often associated with GB diffusion and shuffling of atom inside GB. One scenario is that the dislocation emitted from a GB, traveled through the entire grain, and wash eventually absorbed in the opposite GB [34]. The other scenario is imagined to be that the dislocation bows out to a semicircle from the abundant GB source and injects a lattice dislocation at a relative low stress [35]. Meanwhile, the crack-induced stress concentration was also in support of dislocation emission at a GB facet [36], which may also induce a low nucleation stress for GB dislocation. The enhanced GB process associated with dislocation activation may be responsible for reduced hardness with decreasing *d*. A model of "grain boundary-affected zone" at and near the GB was proposed to explain the enhanced rate sensitivity of NC Ni [11]. The MD simulation indicates that the atoms at GB are easier to deform than that inside the grain for NC Cu and Ni [37,38]. For the present NC Ta films, the volume percentage of the GB increases from 27.1 to 48.8 vol% as *d *decreases from 20 to 10 nm. In both cases, the volume percentage of the GB is much higher than that of the UFG/CG metals. Therefore, it is believed that the enhanced GB-mediated processes involving atomic diffusion and dislocation generation at GB are responsible for the decreased hardness and increased rate sensitivity with reduced *d*.

## Conclusions

In summary, we have examined the rate sensitivity and hardness of NC tetragonal Ta films by indentation creep and loading rate change tests. It is suggested that *m*_l _and *m*_c _increase with the decrease of *d *and *h*, respectively, which exhibits a remarkable size effect. The hardness becomes smaller as *d *decreases from 20 to 10 nm. The Coble creep and GB sliding are excluded for dominant deformation mechanism. Instead, GB activation processes involving atomic diffusion and dislocation generation at GB are enhanced to mediate the plastic deformation process.

## Abbreviations

CG: coarse grain; FWHM: full widths at half maximum; GB: grain boundary; NC: nanocrystalline; TEM: transmission electron microscopy; UFG: ultrafine grain; XRD: X-ray diffraction.

## Competing interests

The authors declare that they have no competing interests.

## Authors' contributions

CZH designed the project of experiment, carried out the preparation of Ta films, and drafted the manuscript. SQW performed microstructure characterization including in XRD and TEM. HYL performed nanoindentation testing. MXK participated in the design of the study and revised the manuscript.
